# Delirium is more common and associated with worse outcomes in Parkinson’s disease compared to older adult controls: results of two prospective longitudinal cohort studies

**DOI:** 10.1093/ageing/afae046

**Published:** 2024-03-15

**Authors:** Florence Gerakios, Alison J Yarnall, Gemma Bate, Laura Wright, Daniel Davis, Blossom C M Stephan, Louise Robinson, Carol Brayne, Glenn Stebbins, John-Paul Taylor, David J Burn, Louise M Allan, Sarah J Richardson, Rachael A Lawson

**Affiliations:** Translational and Clinical Research Institute, Newcastle University, Newcastle upon Tyne, UK; Newcastle upon Tyne NHS Foundation Trust, Newcastle upon Tyne, UK; Translational and Clinical Research Institute, Newcastle University, Newcastle upon Tyne, UK; Newcastle upon Tyne NHS Foundation Trust, Newcastle upon Tyne, UK; NIHR Newcastle Biomedical Research Centre, Faculty of Medical Sciences, Newcastle University, UK; Translational and Clinical Research Institute, Newcastle University, Newcastle upon Tyne, UK; Translational and Clinical Research Institute, Newcastle University, Newcastle upon Tyne, UK; MRC Unit for Lifelong Health and Ageing, University College London, London, UK; Institute of Mental Health, School of Medicine, Nottingham University, Nottingham, UK; Dementia Centre of Excellence, EnAble Institute, Curtin University, Perth, Australia; Population Health Sciences Institute, Faculty of Medical Sciences, Newcastle University, Newcastle upon Tyne, UK; Department of Psychiatry, University of Cambridge, Cambridge, UK; Department of Neurological Sciences, Rush University Medical Center, Chicago, USA; Translational and Clinical Research Institute, Newcastle University, Newcastle upon Tyne, UK; NIHR Newcastle Biomedical Research Centre, Faculty of Medical Sciences, Newcastle University, UK; Translational and Clinical Research Institute, Newcastle University, Newcastle upon Tyne, UK; Centre for Research in Ageing and Cognitive Health, University of Exeter, Exeter, UK; Translational and Clinical Research Institute, Newcastle University, Newcastle upon Tyne, UK; NIHR Newcastle Biomedical Research Centre, Faculty of Medical Sciences, Newcastle University, UK; Translational and Clinical Research Institute, Newcastle University, Newcastle upon Tyne, UK; NIHR Newcastle Biomedical Research Centre, Faculty of Medical Sciences, Newcastle University, UK

**Keywords:** Delirium, Parkinson's disease, Outcomes, Mortality, Institutionalisation, Older people

## Abstract

**Background:**

Inpatient prevalence of Parkinson’s disease (PD) delirium varies widely across the literature. Delirium in general older populations is associated with adverse outcomes, such as increased mortality, dementia, and institutionalisation. However, to date there are no comprehensive prospective studies in PD delirium. This study aimed to determine delirium prevalence in hospitalised PD participants and the association with adverse outcomes, compared to a control group of older adults without PD.

**Methods:**

Participants were hospitalised inpatients from the ‘Defining Delirium and its Impact in Parkinson’s Disease’ and the ‘Delirium and Cognitive Impact in Dementia’ studies comprising 121 PD participants and 199 older adult controls. Delirium was diagnosed prospectively using the Diagnostic and Statistical Manual of Mental Disorders 5^th^ Edition criteria. Outcomes were determined by medical note reviews and/or home visits 12 months post hospital discharge.

**Results:**

Delirium was identified in 66.9% of PD participants compared to 38.7% of controls (p < 0.001). In PD participants only, delirium was associated with a significantly higher risk of mortality (HR = 3.3 (95% confidence interval [CI] = 1.3–8.6), p = 0.014) and institutionalisation (OR = 10.7 (95% CI = 2.1–54.6), p = 0.004) 12 months post-discharge, compared to older adult controls. However, delirium was associated with an increased risk of developing dementia 12 months post-discharge in both PD participants (OR = 6.1 (95% CI = 1.3–29.5), p = 0.024) and in controls (OR = 13.4 (95% CI = 2.5–72.6), p = 0.003).

**Conclusion:**

Delirium is common in hospitalised PD patients, affecting two thirds of patients, and is associated with increased mortality, institutionalisation, and dementia. Further research is essential to understand how to accurately identify, prevent and manage delirium in people with PD who are in hospital.

## Key Points

- Delirium occurred in two thirds of people with Parkinson’s disease when hospitalised.
- People with Parkinson’s disease are much more likely to get delirium whilst in hospital than older adults without Parkinson’s disease (67% vs. 39%).
- An episode of delirium whilst in hospital increases the risk of mortality, institutionalisation, and dementia in Parkinson’s disease.
- The risks of mortality and institutionalisation following delirium are higher in those with Parkinson’s disease than older adults without Parkinson’s disease.

## Background

Delirium is a serious, neuropsychiatric syndrome defined by acute changes in attention, level of arousal and cognition [1]. Delirium is often both underreported and under recognised [2–4]. Parkinson’s disease (PD) may be a risk factor for delirium, but reported inpatient prevalence varies from 11–60% [5]. This variance is likely due to challenges in recognising delirium in PD due to shared clinical features [6], the range of criteria and methods used for delirium diagnosis [5], along with the reliance on retrospective delirium ascertainment in many studies, which has been shown to miss nearly two thirds of cases of delirium in PD [7].

In older adults, delirium has been associated with increased risk of institutionalisation, dementia, and mortality [8–10]. However, there is a paucity of similar evidence in PD. One recent retrospective study of people with PD found that delirium was associated with a three-fold increase in mortality and in those with PD or a parkinsonism it was associated with a six-fold increased risk of dementia [11]. However, to date, there are no comprehensive prospective studies that use robust definitions of delirium with longitudinal follow up. This study aimed to comprehensively determine the prevalence of delirium and its association with mortality, institutionalisation, and dementia at 12 months in an inpatient population with PD compared to older adults without PD.

## Methods

### Population

Participants were from the ‘Defining Delirium and its Impact in Parkinson’s Disease’ (DELIRIUM-PD) and the ‘Delirium and Cognitive Impact in Dementia’ (DECIDE) [12] studies comprising participants with PD and an older adult, non-PD control group. The DELIRIUM-PD study included people with PD diagnosed by a movement disorders expert using the Queen’s Square Brain Bank Criteria [13] who attended outpatient clinics in Newcastle Hospitals for the management of their PD. Participants were invited to take part on admission to hospitals in Newcastle between 7^th^ March 2018 and 31st January 2022 (the study was paused due to the COVID-19 pandemic during the periods of 17th March 2020 to 5th October 2020, 5th January 2021 to 2nd March 2021 and 7th January 2022 to the end of the recruitment period).

The DECIDE study (general older adults which included people with multimorbidity, such as PD or dementia) was nested within the Cognitive Function and Ageing Study II—Newcastle cohort (CFAS II–Newcastle) [14]. CFAS II-Newcastle is a large, population-based cohort of people aged≥65 years from three geographical areas in the UK including Newcastle upon Tyne, measuring prevalence and incidence of dementia. Parkinson’s disease participants had a diagnosis based on criteria from the Queen’s Square Brain Bank [13]. From 5th January 2016 to 5th January 2017, participants from CFAS II-Newcastle were invited to participate in DECIDE on admission to hospitals in Newcastle. In both studies, elective and emergency admissions, in both surgical and medical settings, were included.

In both studies, potential participants were provided with written information and given the opportunity to opt-out of further contact. An electronic recurring admission patient alert (RAPA) was added to their records to alert researchers when they were admitted to either the Royal Victoria Infirmary (RVI) or Freeman Hospital (FRH), Newcastle upon Tyne, UK. For each study, the research team aimed to approach participants as soon as possible following admission.

These studies were approved by Research Ethics Committees (DELIRIUM-PD: Yorkshire & The Humber—Bradford Leeds Research Ethics Committee 18/YH/0486; DECIDE: Newcastle and North Tyneside 2 Regional Ethics Committee 15/NE/0353). A formal capacity assessment based on the Mental Capacity Act [15] was performed by a trained member of the research team. If participants lacked capacity to provide full written informed consent, a personal consultee was identified. They provided advice on participation as per Section 32 of the Mental Capacity Act [15].

Participants were excluded from the studies if they lacked capacity to give informed consent and did not have an appropriate consultee available, if they were receiving end of life care, they were isolated for infection control, or they were expected to be in hospital for fewer than 24 hours. In the DELIRIUM-PD study, participants were additionally excluded if they had a diagnosis of non-idiopathic PD or atypical parkinsonian disorders, they were not recruited within 72 hours of admission or had insufficient command of the English language to take part in the cognitive assessments.

### Data collection

In both studies, baseline demographic and clinical data were collected at recruitment on admission to hospital, including age, sex, comorbidities, medications, pre-admission frailty (using the Clinical Frailty Scale, CFS [16]), living arrangements and functional dependency. In DELIRIUM-PD, participants with mild cognitive impairment (PD-MCI) or PD dementia (PDD) were identified from clinic letters by their treating clinician. In DECIDE, baseline dementia diagnosis was ascertained from the interview for CFAS II using an algorithmic approach called the Automated Geriatric Examination for Computer Assisted Taxonomy [17]. The full content of the interviews is available online (http://www.cfas.ac.uk). In DELIRIUM-PD, PD duration, levodopa equivalent daily dose (LEDD) [18], motor severity as measured by the total Movement Disorder Society—Unified Parkinson’s Disease Rating Scale Part III (MDS-UPDRS III) score and the Hoehn and Yahr stage were also assessed [18].

### Delirium ascertainment

Delirium was assessed using a structured interview based upon the Diagnostic and Statistical Manual of Mental Disorders 5^th^ Edition (DSM-5) criteria as described previously [12]. In brief, this comprised researcher observations, standardised testing and information obtained from the informant and hospital staff to consider changes in attention, level of arousal and cognition and to establish an acute change from baseline (Supplementary Table 1) [19–21]. When there was diagnostic uncertainty, vignettes were presented to an expert consensus panel made up of two expert independent reviewers and a third for any disagreement of cases (DELIRIUM-PD: FG and RAL, and AJY or SJR; DECIDE: LMA and DHJD, SGP) [22]. Delirium screening was performed consecutively for up to five days where possible. Following this, participants were seen once weekly until discharge (DELIRIUM-PD), or daily until delirium resolution or screened twice weekly (DECIDE).

**Table 1:** Baseline demographics and clinical characteristics comparing PD and controls with and without delirium

Variable	PD (n = 121)		Controls (n = 199)				
	Delirium n = 81	No Delirium n = 40	Delirium n = 77	No Delirium n = 122	F/Z/χ^2^	p-value	Pairwise
Age (years)	78(9)	74.5(17.5)	85(9)	81(9.5)	χ^2^ = 52.8	**<0.001**	**b,d,e,f**
Women, n(%)	29(35.8)	18(45)	39(50.6)	68(55.7)	χ^2^ = 8.1	**0.044**	**c**
Years in education	11(3)	11(2.8)	10(1)	10(2)	χ^2^ = 43.4	**<0.001**	**b,c,d,f**
Clinical Frailty Scale	6(2)	5(2)	5(2)	4(2)	χ^2^ = 102.8	**<0.001**	**a,b,c,e,f**
Number of Admissions	2(1.5)	1(.00)	2(1.5)	1(1)	χ^2^ = 16.8	**<0.001**	**a, d**
PD duration (months)	69(83)	97(110)			U = 1,183	0.077	
LEDD mg/day	600(475)	600(641.25)			U = 1,488	0.605	
MDS-UPDRS III Total Score^*^	62.5(24.5)	50(13)			U = 765	**0.003**	
Cognition							
No cognitive impairment, n(%)	38(46.9)	30(75.0)	62(80.5)	118(96.7)	χ^2^ = 69.9	**<.001**	**a, b, c, e, f**
PD-MCI, n(%)	24(29.6)	7(17.5)			χ^2^ = 2.1	0.187	
Dementia, n(%)	19(23.5)	3(7.5)	15(19.5)	4(3.3)	χ^2^ = 22.2	**<.001**	**c, e**
Care Home, n(%)	8(9.9)	1(2.5)	5(6.5)	6(4.9)	χ^2^ = 3.2	0.368	

All are median and IQR unless stated otherwise. Significant results highlighted in bold. ^*^Missing in 21 participants. Pair-wise comparisons – significant results after correction for multiple comparisons p < 0.008. A = PD Delirium vs PD No Delirium, B=PD Delirium vs Control Delirium, C=PD Delirium vs Control No Delirium, D=PD No Delirium vs Control Delirium, E = Control Delirium vs Control No Delirium, F=Control No Delirium vs PD No Delirium

Parkinson’s disease (PD); Parkinson’s disease Mild Cognitive Impairment (PD-MCI); Parkinson’s disease Dementia (PDD); Levodopa Equivalent Daily Dose (LEDD); Movement Disorder Society—Unified Parkinson’s disease rating Scale (MDS-UPDRS)

### Outcomes

#### Death

Both studies determined date of death up to 12 months following last hospital discharge by review of medical records.

#### Dementia

In DELIRIUM-PD, medical notes were reviewed and a new diagnosis of PDD was determined through clinicians’ diagnosis and agreed expert consensus (RAL, FG and AJY) based on MDS diagnostic criteria, in keeping with previous work [23, 24]. This comprised evidence of cognitive decline, indicated by impaired cognitive screening tools, subjective cognitive decline and/or impaired functional dependence [25–27]. Dementia diagnosis was collected 12 months after the last dementia free discharge.

In DECIDE, a home visit was carried out 12 months after the most recent hospital discharge, to repeat the CFAS II interview performed at baseline, described above.

#### Institutionalisation

In DELIRIUM-PD, new institutionalisation was collected 12 months post last discharge compared to where participants lived at baseline.

In DECIDE, a home visit was carried out 12 months after the most recent hospital discharge, and institutionalisation was determined as where they lived during this follow up visit compared to where they lived at baseline.

### Statistical analysis

Statistical analysis was completed in SPSS Statistics (Version 28; SPSS Armonk, NY: IBM Corp) and using R software (Version 4.2.1; R Foundation for Statistical Computing, Vienna, Austria) implementing *Survival* [28], *survminer* [29], *ggplot2* [30]*,* and *ggpubr* [31] packages. Participants from each study were classified as having PD or as controls (older adults without PD); all participants including those with baseline cognitive impairment were included in these analyses. Normality of continuous variables was tested using the Kolmogorov–Smirnov statistic and visual inspections of histograms. Chi-squared, Kruskal-Wallis or Mann–Whitney U tests were implemented, as appropriate, to compare baseline group differences. Survival time and cumulative survival were calculated using the date of the last hospital discharge to date of death or censored at approximately 12 months post discharge using Kaplan–Meier plots. Multivariate Cox regression was used to determine whether delirium was associated with survival in PD participants and controls. Multivariate binomial logistic regression models were used to determine whether delirium was associated with experiencing at least one outcome, a new diagnosis of dementia or new institutionalisation in surviving PD and control participants. For these analyses, those with baseline dementia or institutionalisation were excluded from models evaluating risk of new dementia or institutionalisation, respectively. Covariates included age, sex, and frailty in all models; additionally baseline dementia was included for death and institutionalisation.

## Results

### Characteristics

**Figure 1:**
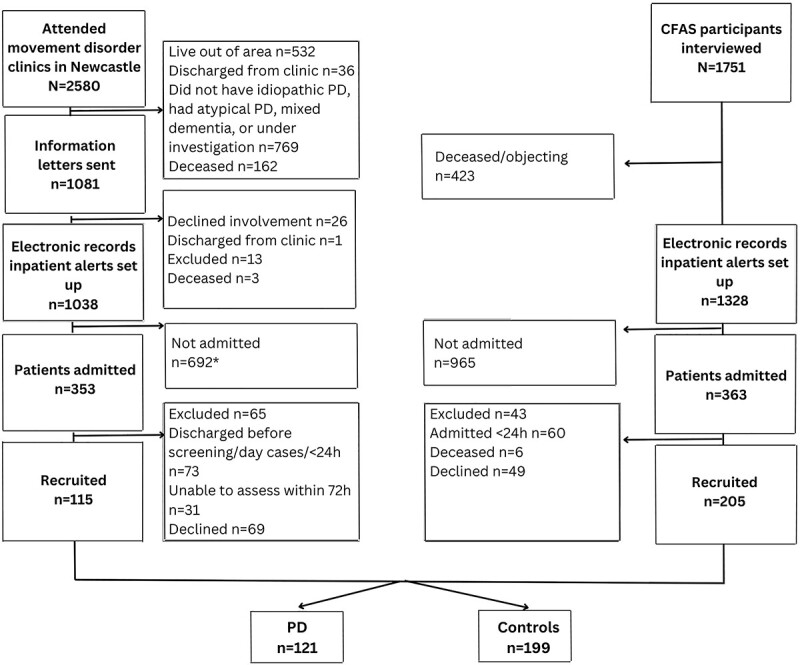
Recruitment flowchart for DELIRIUM-PD. ^*^Does not include people with PD admitted to hospital during study pauses due to COVID-19 pandemic Parkinson’s disease (PD); Delirium and Cognitive Impact in Dementia (DECIDE); Defining Delirium and its Impact in Parkinson’s disease (DELIRIUM-PD).

Participants from the DELIRIUM-PD study (n = 115) and the DECIDE study (n = 205) comprised: 121 participants with PD and 199 older adult controls. Participants were excluded on admission if they had an atypical parkinsonism (DELIRIUM-PD n = 15) or inappropriate diagnosis (DECIDE n = 12), being near death or too unwell (DELIRIUM-PD n = 20; DECIDE n = 0), having no informant available (DELIRIUM-PD n = 5; DECIDE n = 0), lacking capacity with no appropriate consultee (DELIRIUM-PD n = 11; DECIDE n = 12), having insufficient English to complete assessments (DELIRIUM-PD n = 6), having an infectious disease (DELIRIUM-PD n = 7; DECIDE n = 5), being out of area (DELIRIUM-PD n = 1), or not approached (DECIDE n = 14) (Figure 1). There were 173 participants with outcome data available from DECIDE and 115 from DELIRIUM-PD. PD participants were significantly younger than controls (median = 77 years, IQR = 11 *vs.* median = 82 years, IQR = 9, respectively), had more years in education (median = 11 years, IQR = 3 *vs.* median = 10 years, IQR = 2, respectively) and were frailer (median CFS = 6, IQR = 1 *vs.* median CFS = 4, IQR = 2 respectively, p < 0.001 for all). Of the PD participants, 18.2% (n = 22) had a dementia on admission to hospital compared to 9.5% (n = 19) of controls (p = 0.020). Delirium occurred in 66.9% (n = 81) of participants with PD compared to 38.7% of older adult controls (n = 77, p < 0.001). Parkinson’s disease participants with delirium were frailer compared to those without delirium (CFS median = 6, IQR = 2 *vs.* median = 5, IQR = 2, respectively, p < 0.001), had more severe motor symptoms (MDS-UPDRS III median = 62.5, IQR = 24.5*vs.* median = 50, IQR = 13, respectively, p = 0.003) and had more hospital admissions (median = 2, IQR = 1.5 *vs.* median = 1, IQR = 0.00, respectively, p < 0.001).

### Mortality

**Figure 2:**
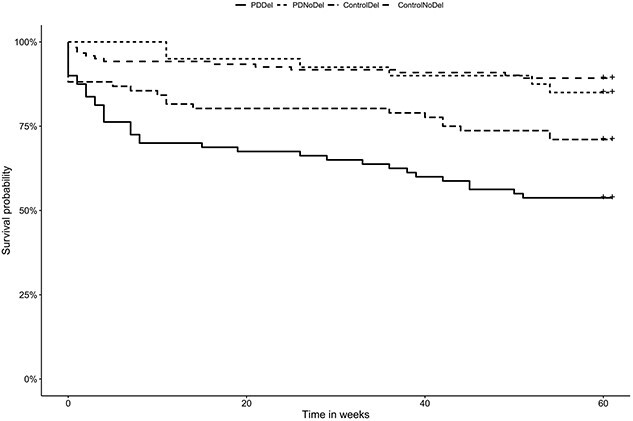
A Kaplan–Meier survival curve in PD participants and controls with and without delirium.

In PD participants with delirium, 46.9% (n = 38/81) died within 12 months of their last discharge compared to 15% (n = 6/40) of participants with PD without delirium (p < 0.001) and 29.9% (n = 23/77) of the control participants with delirium (p = 0.021). PD participants with delirium had a significantly lower cumulative survival probability at 12 months compared to those PD participants without delirium (53.7% *vs.* 85%, respectively, p < 0.001) and to controls with delirium (53.7% *vs.* 71.1%, p = 0.025, Figure 2). Controls with delirium had a significantly lower cumulative survival probability at 12 months compared to controls without delirium (71.1% *vs.* 89.3%, p < 0.001). Participants with PD delirium had three times the risk of mortality (HR = 3.3, p = 0.014) at 12 months post discharge compared to those without delirium, adjusted for age, sex, frailty and baseline dementia. Whereas mortality risk did not significantly differ in controls with and without delirium (p > 0.05, Table 2).

**Table 2:** Delirium as an independent predictor of death, dementia diagnosis, and institutionalisation.

	PD	Control
Death^a^^*^	3.3 (1.3–8.6), **p = 0.014**	2.2 (1.0–4.7), p = 0.053
New dementia^b^	6.1 (1.3–29.5), **p = 0.024**	13.4 (2.5–72.6), **p = 0.003**
New institutionalisation^a^	10.4 (2.0–52.9), **p = 0.005**	5.4 (.5–60.7), p = 0.170
Any adverse outcome^b^	4.7 (1.8–12.1), **p = 0.001**	6.8 (2.7–14.2), **p < 0.001**

Data presented are odds ratio (95% confidence interval), *P* value unless otherwise stated. Significant results highlighted in bold. PD—Parkinson’s disease.

^^*^^Cox regression. Data presented are hazard ratio (95% confidence interval), *P* value.

Covariates included in model are a: age (years), sex, binary clinical frailty with cut point 5, baseline dementia; b: age, sex, binary clinical frailty.

### Dementia

Of the surviving participants, who were dementia free at admission, 37.2% (n = 16/43) of those with PD and delirium developed dementia within 12 months compared to 9.4% (3/32) of those without delirium (p = 0.005) and 27% (n = 10/37) of the control delirium group (p = 0.233). Delirium increased the risk of developing dementia by six times (OR = 6.1) in PD participants compared to 13 times (OR = 13.4) in controls (p < 0.05 for both, Table 2) adjusted for age, sex, and frailty.

### Institutionalisation

48.1% (n = 25/52) of surviving PD participants with delirium who were not in care at baseline were institutionalised within 12 months compared to 5.9% (2/34) of those without delirium (p < 0.001) and 16.3% (7/43) of the control delirium group (p = 0.002). Delirium incurred ten times (OR = 10.4, p = 0.005) increased risk of institutionalisation in the PD group but there was no significant risk in the control group (p > 0.05, table 2) adjusted for age, sex, frailty, and baseline dementia.

### Any adverse outcome

Most of those with PD and delirium (70%, n = 56/81) experienced at least one adverse outcome (comprising death, dementia, or institutionalisation) compared to 27.5% (n = 11/40) of those without delirium (p < 0.001) and 58.8% (n = 40/68) of the control delirium group (p < 0.001). Delirium increased the risk of a negative outcome by four-fold in PD participants and six-fold in controls (OR = 4.7 and OR = 6.8, respectively, p < 0.01 for both, Table 2).

## Discussion

In the first prospective study of delirium in PD, we have shown that two-thirds of people with PD develop delirium during hospitalisation compared to just over one third of older adults without PD. We have also shown that delirium in PD is associated with significantly higher risks of institutionalisation and death within one year, compared to delirium in older adults without PD. Taken together, these results show that the high prevalence and poor outcomes seen with delirium appear to be exaggerated in PD.

When compared to existing literature, our delirium prevalence of 67% in people with PD in hospital was higher (22–48%) than reported previously in medical PD inpatients [5]. It is likely that published studies have underestimated delirium prevalence due to several methodological limitations. First, the methods used for delirium detection were highly heterogeneous, including DSM-IV criteria, a new prescription of antipsychotic medications and the use of delirium screening tools, none of which have been validated in PD [5]. Second, multiple studies have relied on retrospective medical notes reviews to diagnose delirium, despite delirium in PD often being underreported in patient medical notes [7]. In older adults without PD, it is well established that delirium is associated with increased mortality [32–35]. However, no previous prospective studies have specifically aimed to evaluate outcomes after delirium in PD. A retrospective study of outcomes in a PD cohort of 191 showed a three-fold increased risk of mortality over a 16 year period [11]. We also found a three-fold increased risk of mortality in people with PD who experienced delirium but within a much shorter follow up, with only just over half of patients with PD surviving to one year after an episode of delirium. Another small study found survival rates to be worse over a five-year period in a Parkinsonian delirium group compared to a control group [36]. Our study built on this by directly comparing survival rates following delirium in people with and without PD, demonstrating the immediacy and urgency of the outcome and reporting a more accurate risk estimate by using a much larger cohort. Whilst controls with delirium had a lower cumulative survival probability than controls without delirium, this association did not remain significant when controlling for age, sex, clinical frailty, and baseline dementia. We have not only confirmed the risk of mortality within the PD population, but we have also shown for the first time that PD increases the risk of mortality over and above a delirium in older adults.

Whilst as far as we are aware, the risk of institutionalisation following an episode of delirium has never previously been considered in a PD cohort, several studies have shown that delirium increases the risk of institutionalisation by up to three times in general older adult populations [10]. We showed that people with PD who experienced delirium had a 10-fold increased risk of institutionalisation within a year even when controlling for age, sex, frailty, and baseline dementia. There are several potential reasons for this. Hallucinations are often a predominant issue leading to institutionalisation in PD, especially when considering the associated carer burden [37]. Functional decline is also a driver of nursing home placement [38]. Both may be exacerbated following delirium [3] and may explain this increased risk of nursing home placement. Understanding the magnitude and drivers underlying this increased risk is vital on both an individual and population level, particularly given the current crisis in social care.

We found that participants with PD were at a six-fold increased risk of developing dementia following their delirium compared to PD participants without delirium. This is in line with results from a retrospective outcomes study by Green et al [11]. We found that the risk of dementia was lower in PD compared to our older adult controls without PD. This may be due to considerable survival bias with the higher mortality in the PD group (46.9%) compared to the control group (29.9%); it is possible that a greater proportion of PD participants may have developed PDD had they survived. We found that participants with PD were at an increased risk of mortality, institutionalisation, and dementia, but in controls, delirium was only a predictor of dementia. This raises the question of whether there is something phenotypically or mechanistically different to PD related delirium compared to older adults without PD. Although exploring this was outside the scope of this study, speculatively, deficits in neurotransmitters (e.g. dopamine and acetylcholine) and proinflammatory cytokine profiles associated with PD may play a role [3, 39]. It is important for future research to consider if there is a phenotypic reason for this, or if the outcomes are influenced by other factors related to PD, such as disease severity or clinical frailty.

A major strength of this work was the combining of two prospective studies using a standardised approach to delirium ascertainment, allowing a direct comparison between PD and older adult cohorts over time. However, data collection was at one time point each day and frequency of delirium screening differed between studies. In DELIRIUM-PD, there was no data collection on weekends or bank holidays and visits were reduced to once weekly after the five consecutive days. Although this was mitigated by reviewing medical notes and gaining collateral history from family members and hospital staff, it is possible that some episodes of delirium were missed due to the fluctuating nature of the condition. Due to the complex nature of delirium and hospital admissions in older adults, we were unable to account for differences in acute disease severity, prescribed medications, history of falls and admission type in this analysis. These may have had an impact on delirium prevalence and adverse outcomes. However, the aetiologies and driving factors of delirium are extensive, where the cause can often be multifactorial and subtle, making it difficult to quantify. The scale of the problem in terms of identifying causes of delirium has been demonstrated in a recent systematic review which reported 112 precipitating and predisposing factors including systemic illness, metabolic abnormality, surgical and pharmacological factors [40]. Despite this, it does highlight the need for delirium to be assessed. During the COVID-19 pandemic (2020–2022), there was a reduction in in-person clinic visits during the DELIRIUM-PD study period and, consequently, dementia cases may have been underestimated. We used a robust consensus criterion for dementia diagnosis alongside medical notes reviews to manage this and the differing methods for identifying dementia across the studies. Finally, PD participants were frailer than controls at baseline. Although frailty was a covariate in all models, it is difficult to disentangle the relationship between frailty and delirium, with poor outcomes. Whilst we may expect worse clinical frailty to be a driving factor of survival rates, when considering the bidirectional relationship between the risk of delirium and frailty, along with the prevalence within a PD population, it is a complex area where further research is essential [41–44]. Our work has highlighted the necessity to prevent and manage delirium in PD, particularly when considering that delirium can be preventable in some cases [45]. However, there is currently no PD-specific evidence available to implement this change [6]. A major challenge is differentiating delirium from PD due to the overlap in clinical features. Despite this, only 11 studies pertain to delirium symptoms in PD participants, no studies attempt to differentiate PD symptoms from acute symptoms of delirium and, there are no studies that have investigated PD in delirium longitudinally [5, 6]. For clinicians to know how to accurately identify, prevent and manage delirium, future work must first describe the phenomenology of delirium in PD, taking into consideration its subtypes. Subsequently, delirium screening tools may need to be altered to be more suitable for a PD delirium diagnosis [46].

In conclusion, people with PD are much more likely to develop delirium in hospital than older adults without PD. Delirium in PD leads to an increased risk of death, dementia, and institutionalisation. There is an urgent need for further research in people with PD to determine how best to diagnose and manage delirium, including clinical trials to reduce the risk of adverse outcomes.

## Supplementary Material

aa-23-1348-File004_afae046

## References

[1] Wilson JE , Mart MF, Cunningham C et al. Delirium. Nat Rev Dis Primers 2020; 6: 90. 33184265 10.1038/s41572-020-00223-4 PMC9012267

[2] Oh ES , Fong TG, Hshieh TT, Inouye SK. Delirium in older persons: advances in diagnosis and treatment. JAMA 2017; 318: 1161–74. 28973626 10.1001/jama.2017.12067 PMC5717753

[3] Vardy ER , Teodorczuk A, Yarnall AJ. Review of delirium in patients with Parkinson's disease. J Neurol 2015; 262: 2401–10. 25957635 10.1007/s00415-015-7760-1

[4] Geriatric Medicine Research C . Delirium is prevalent in older hospital inpatients and associated with adverse outcomes: results of a prospective multi-Centre study on world delirium awareness day. BMC Med 2019; 17: 229. 31837711 10.1186/s12916-019-1458-7 PMC6911703

[5] Lawson RA , McDonald C, Burn DJ. Defining delirium in idiopathic Parkinson's disease: a systematic review. Parkinsonism Relat Disord 2019; 64: 29–39. 30279060 10.1016/j.parkreldis.2018.09.025

[6] Ebersbach G , Ip CW, Klebe S et al. Management of delirium in Parkinson's disease. J Neural Transm (Vienna) 2019; 126: 905–12. 30725186 10.1007/s00702-019-01980-7

[7] Cullinan RJ , Richardson SJ, Yarnall AJ, Burn DJ, Allan LM, Lawson RA. Documentation and diagnosis of delirium in Parkinson's disease. Acta Psychiatr Scand 2022; 147: 527–35. 35771186 10.1111/acps.13470 PMC10952625

[8] Richardson SJ , Davis DHJ, Stephan BCM et al. Recurrent delirium over 12 months predicts dementia: results of the delirium and cognitive impact in dementia (DECIDE) study. Age Ageing 2021; 50: 914–20. 33320945 10.1093/ageing/afaa244 PMC8099011

[9] Tieges Z , Quinn T, MacKenzie L et al. Correction to: association between components of the delirium syndrome and outcomes in hospitalised adults: a systematic review and meta-analysis. BMC Geriatr 2021; 21: 490. 34503451 10.1186/s12877-021-02419-z PMC8427852

[10] Tachibana M , Inada T. Poor prognostic impact of delirium: especially on mortality and institutionalisation. Psychogeriatrics 2023; 23: 187–95. 36416212 10.1111/psyg.12914

[11] Green S , Perrott SL, McCleary A et al. First delirium episode in Parkinson's disease and parkinsonism: incidence, predictors, and outcomes. NPJ Parkinsons Dis 2021; 7: 92. 34635668 10.1038/s41531-021-00234-2 PMC8505483

[12] Richardson SJ , Davis DHJ, Stephan B et al. Protocol for the delirium and cognitive impact in dementia (DECIDE) study: a nested prospective longitudinal cohort study. BMC Geriatr 2017; 17: 98. 28454532 10.1186/s12877-017-0479-3 PMC5410072

[13] Hughes AJ , Daniel SE, Kilford L, Lees AJ. Accuracy of clinical diagnosis of idiopathic Parkinson's disease: a clinico-pathological study of 100 cases. J Neurol Neurosurg Psychiatry 1992; 55: 181–4. 1564476 10.1136/jnnp.55.3.181 PMC1014720

[14] Davis DH , Barnes LE, Stephan BC et al. The descriptive epidemiology of delirium symptoms in a large population-based cohort study: results from the Medical Research Council cognitive function and ageing study (MRC CFAS). BMC Geriatr 2014; 14: 87. 25066510 10.1186/1471-2318-14-87 PMC4126352

[15] GREAT . BRITAIN Mental Capacity Act 2005, 2005.

[16] Sternberg SA , Wershof Schwartz A, Karunananthan S et al. The identification of frailty: a systematic literature review. J Am Geriatr Soc 2011; 59: 2129–38. 22091630 10.1111/j.1532-5415.2011.03597.x

[17] Matthews FE , Arthur A, Barnes LE et al. A two-decade comparison of prevalence of dementia in individuals aged 65 years and older from three geographical areas of England: results of the cognitive function and ageing study I and II. Lancet 2013; 382: 1405–12. 23871492 10.1016/S0140-6736(13)61570-6 PMC3906607

[18] Schade S , Mollenhauer B, Trenkwalder C. Levodopa equivalent dose conversion factors: an updated proposal including Opicapone and safinamide. Mov Disord Clin Pract 2020; 7: 343–5. 32258239 10.1002/mdc3.12921 PMC7111582

[19] Tieges Z , McGrath A, Hall RJ, Maclullich AM. Abnormal level of arousal as a predictor of delirium and inattention: an exploratory study. Am J Geriatr Psychiatry 2013; 21: 1244–53. 24080383 10.1016/j.jagp.2013.05.003

[20] Chester JG , Beth Harrington M, Rudolph JL, Group VADW. Serial administration of a modified Richmond agitation and sedation scale for delirium screening. J Hosp Med 2012; 7: 450–3. 22173963 10.1002/jhm.1003 PMC4880479

[21] American Psychiatric Association D, Association AP. Diagnostic and Statistical Manual of Mental Disorders: DSM-5. American psychiatric association Washington, DC, 2013.

[22] Kuhn E , Du X, McGrath K et al. Validation of a consensus method for identifying delirium from hospital records. PloS One 2014; 9: e111823. 25369057 10.1371/journal.pone.0111823 PMC4219785

[23] Goetz CG , Emre M, Dubois B. Parkinson's disease dementia: definitions, guidelines, and research perspectives in diagnosis. Ann Neurol 2008; 64 Suppl 2: S81–92. 19127578 10.1002/ana.21455

[24] Lawson RA , Williams-Gray CH, Camacho M et al. Which neuropsychological tests? Predicting cognitive decline and dementia in Parkinson's disease in the ICICLE-PD cohort. J Parkinsons Dis 2021; 11: 1297–308. 34024781 10.3233/JPD-212581 PMC8461722

[25] Jorm AF . The informant questionnaire on cognitive decline in the elderly (IQCODE): a review. Int Psychogeriatr 2004; 16: 275–93. 15559753 10.1017/s1041610204000390

[26] Nasreddine ZS , Phillips NA, Bedirian V et al. The Montreal cognitive assessment, MoCA: a brief screening tool for mild cognitive impairment. J Am Geriatr Soc 2005; 53: 695–9. 15817019 10.1111/j.1532-5415.2005.53221.x

[27] Emre M , Aarsland D, Brown R et al. Clinical diagnostic criteria for dementia associated with Parkinson's disease. Mov Disord 2007; 22: 1689–707 quiz 1837. 17542011 10.1002/mds.21507

[28] Therneau T . A Package for Survival Analysis in R R package version 3.5-3, 2023.

[29] Alboukadel Kassambara MKaPB . In: Rpvs, ed. Survminer: Drawing Survival Curves Using 'ggplot2' 0.4.9, 2021.

[30] Wickham H . ggplot2: Elegant Graphics for Data Analysis York S-VNs, 2016.

[31] Alboukadel Kassambara MKaPB . Ggpubr: 'ggplot2' Based Publication Ready Plots 0.5.0 Rpvs (ed), 2022.

[32] Eeles EM , Hubbard RE, White SV, O'Mahony MS, Savva GM, Bayer AJ. Hospital use, institutionalisation and mortality associated with delirium. Age Ageing 2010; 39: 470–5. 20554540 10.1093/ageing/afq052

[33] McCusker J , Cole M, Abrahamowicz M, Primeau F, Belzile E. Delirium predicts 12-month mortality. Arch Intern Med 2002; 162: 457–63. 11863480 10.1001/archinte.162.4.457

[34] Salluh JI , Wang H, Schneider EB et al. Outcome of delirium in critically ill patients: systematic review and meta-analysis. BMJ 2015; 350: h2538. 26041151 10.1136/bmj.h2538 PMC4454920

[35] Witlox J , Eurelings LS, de Jonghe JF, Kalisvaart KJ, Eikelenboom P, van Gool WA. Delirium in elderly patients and the risk of postdischarge mortality, institutionalization, and dementia: a meta-analysis. JAMA 2010; 304: 443–51. 20664045 10.1001/jama.2010.1013

[36] Serrano-Duenas M , Bleda MJ. Delirium in Parkinson's disease patients. A five-year follow-up study. Parkinsonism Relat Disord 2005; 11: 387–92. 16111911 10.1016/j.parkreldis.2005.05.002

[37] Aarsland D , Larsen JP, Tandberg E, Laake K. Predictors of nursing home placement in Parkinson's disease: a population-based, prospective study. J Am Geriatr Soc 2000; 48: 938–42. 10968298 10.1111/j.1532-5415.2000.tb06891.x

[38] Mueller C , Rajkumar AP, Wan YM et al. Assessment and Management of Neuropsychiatric Symptoms in Parkinson's disease. CNS Drugs 2018; 32: 621–35. 30027401 10.1007/s40263-018-0540-6

[39] Williams-Gray CH , Wijeyekoon R, Yarnall AJ et al. Serum immune markers and disease progression in an incident Parkinson's disease cohort (ICICLE-PD). Mov Disord 2016; 31: 995–1003. 26999434 10.1002/mds.26563 PMC4957620

[40] Ormseth CH , LaHue SC, Oldham MA, Josephson SA, Whitaker E, Douglas VC. Predisposing and precipitating factors associated with delirium: a systematic review. JAMA Netw Open 2023; 6: e2249950. 36607634 10.1001/jamanetworkopen.2022.49950 PMC9856673

[41] McMillan JM , Michalchuk Q, Goodarzi Z. Frailty in Parkinson's disease: a systematic review and meta-analysis. Clin Park Relat Disord 2021; 4: 100095. 34316672 10.1016/j.prdoa.2021.100095 PMC8299963

[42] Persico I , Cesari M, Morandi A et al. Frailty and delirium in older adults: a systematic review and meta-analysis of the literature. J Am Geriatr Soc 2018; 66: 2022–30. 30238970 10.1111/jgs.15503

[43] Zhang XM , Jiao J, Xie XH, Wu XJ. The association between frailty and delirium among hospitalized patients: an updated meta-analysis. J Am Med Dir Assoc 2021; 22: 527–34. 33549566 10.1016/j.jamda.2021.01.065

[44] Quinlan N , Marcantonio ER, Inouye SK, Gill TM, Kamholz B, Rudolph JL. Vulnerability: the crossroads of frailty and delirium. J Am Geriatr Soc 2011; 59: S262–8. 22091571 10.1111/j.1532-5415.2011.03674.x PMC3233987

[45] Siddiqi N , Harrison JK, Clegg A et al. Interventions for preventing delirium in hospitalised non-ICU patients. Cochrane Database Syst Rev 2016; 2016: CD005563. 10.1002/14651858.CD005563.pub3 PMC10431752 26967259

[46] Lawson RA , Richardson SJ, Yarnall AJ, Burn DJ, Allan LM. Identifying delirium in Parkinson disease: a pilot study. Int J Geriatr Psychiatry 2020; 35: 547–52. 31994774 10.1002/gps.5270 PMC7186820

