# Supplementary material for: Delirium is more common and associated with worse outcomes in Parkinson’s disease compared to older adult controls: results of two prospective longitudinal cohort studies
Source: Age Ageing. 2024 Mar 15;53(3):afae046. doi: 10.1093/ageing/afae046 (PMC10945294; doi:10.1093/ageing/afae046)
Supplement: aa-23-1348-File004_afae046 [file aa-23-1348-file004_afae046.docx]

# Supplementary files

**Supplementary Table 1 - DSM-5 diagnostic criteria from the DECIDE study**

| **DSM-5 criteria** | **Test to be performed or information needed** |
| --- | --- |
| **A**. Disturbance in attention (i.e., reduced ability to direct, focus, sustain, and shift attention) and awareness (reduced orientation to the environment). | Observations by the examiner during the interview (initiated by questioning such as “can you tell me what has been going on today?”)  Level of arousal measured using m-RASS and OSLA  Months of the year backwards  Digit Span from MDAS |
| **B**. The disturbance develops over a short period of time (usually hours to a few days), represents a change from baseline attention and awareness, and tends to fluctuate in severity during the course of a day. | Acute onset and/or fluctuation obtained from informant history from nursing staff, next of kin and clinical notes |
| **C**. An additional disturbance in cognition (e.g., memory deficit, disorientation, language, visuospatial ability, or perception). | Impairment in any of the following domains:  SHORT-TERM MEMORY: three item recall at three minutes LONG-TERM MEMORY: when did World War II end?  ORIENTATION: 10 orientation questions from MDAS  LANGUAGE: 3 stage command, naming an object and explain purpose of object along with fluency, comprehension, and content of conversation  VISUOSPATIAL: Will a stone float on water?  PERCEPTUAL DISTURBANCE: evidence of illusions or hallucinations by collateral or direct observation/questioning |
| **D**. The disturbances in criteria A and C are not explained by another pre-existing, established, or evolving neurocognitive disorder and do not occur in the context of a severely reduced level of arousal, such as coma. | Information from history/chart/clinical examination |
| **E.** There is evidence from the history, physical examination, or laboratory findings that the disturbance is a direct physiologic consequence of another medical condition, substance intoxication or withdrawal (i.e., because of a drug of abuse or to a medication), or exposure to a toxin or is because of multiple aetiologies. | Information from history/chart/clinical examination |

Delirium and Cognitive Impact in Dementia (DECIDE) [1]; Modified-Richmond Agitation and Sedation Scale – m-Rass [2]; Observational Scale of Level of Arousal – OSLA [3]; Memoria Delirium Assessment Scale – MDAS [4]; Diagnostic and Statistical Manual of Mental Disorders, Fifth Edition – DSM-5 [5].

References

1 Richardson SJ, Davis DHJ, Stephan BCM, et al. Recurrent delirium over 12 months predicts dementia: results of the Delirium and Cognitive Impact in Dementia (DECIDE) study. *Age Ageing* 2021;50:914-20.

2 Chester JG, Beth Harrington M, Rudolph JL, et al. Serial administration of a modified richmond agitation and sedation scale for delirium screening. *J Hosp Med* 2012;7:450-53.

3 Tieges Z, McGrath A, Hall RJ, et al. Abnormal Level of Arousal as a Predictor of Delirium and Inattention: An Exploratory Study. *The American Journal of Geriatric Psychiatry* 2013;21:1244-53.

4 Lawlor PG, Nekolaichuk C, Gagnon B, et al. Clinical utility, factor analysis, and further validation of the memorial delirium assessment scale in patients with advanced cancer. *Cancer* 2000;88:2859-67.

5 *Diagnostic and statistical manual of Mental disorders: DSM-5*. Washington: American Psychiatric Association, 2013.
